# Genomic analysis based on chromosome-level genome assembly reveals Myrtaceae evolution and terpene biosynthesis of rose myrtle

**DOI:** 10.1186/s12864-024-10509-6

**Published:** 2024-06-10

**Authors:** Ling Yang, Jingjing Jin, Shanwu Lyu, Fangqiu Zhang, Peijian Cao, Qiaomei Qin, Guanghui Zhang, Chen Feng, Peng Lu, Huiguang Li, Shulin Deng

**Affiliations:** 1 Key Laboratory of National Forestry and Grassland Administration On Plant Conservation and Utilization in Southern China & Guangdong Provincial Key Laboratory of Applied Botany, South China Botanical Garden, Chinese Academy of Sciences, Guangzhou, 510650 China; 2 University of Chinese Academy of Sciences, Beijing, 100049 China; 3 National Tobacco Gene Center, Zhengzhou Tobacco Research Institute of CNTC, Zhengzhou, 450001 China; 4 Guangdong Eco-Engineering Polytechnic, Guangzhou, 510520 China; 5 State Key Laboratory of Conservation and Utilization of Bio-Resources in Yunnan & the Key Laboratory of Medicinal Plant Biology of Yunnan Province, Yunnan Agricultural University, Kunming, 650201 China; 6 Jiangxi Provincial Key Laboratory of Ex Situ Plant Conservation and Utilization, Lushan Botanical Garden, Chinese Academy of Sciences, Jiujiang, 332900 China

**Keywords:** *Rhodomyrtus tomentosa*, Genome assembly, *TPS*, Terpene biosynthesis

## Abstract

**Background:**

Rose myrtle (*Rhodomyrtus tomentosa* (Ait.) Hassk), is an evergreen shrub species belonging to the family Myrtaceae, which is enriched with bioactive volatiles (α-pinene and β-caryophyllene) with medicinal and industrial applications. However, the mechanism underlying the volatile accumulation in the rose myrtle is still unclear.

**Results:**

Here, we present a chromosome-level genomic assembly of rose myrtle (genome size = 466 Mb, scaffold N50 = 43.7 Mb) with 35,554 protein-coding genes predicted. Through comparative genomic analysis, we found that gene expansion and duplication had a potential contribution to the accumulation of volatile substances. We proposed that the action of positive selection was significantly involved in volatile accumulation. We identified 43 *TPS* genes in *R. tomentosa*. Further transcriptomic and *TPS* gene family analyses demonstrated that the distinct gene subgroups of *TPS* may contribute greatly to the biosynthesis and accumulation of different volatiles in the Myrtle family of shrubs and trees. The results suggested that the diversity of TPS-a subgroups led to the accumulation of special sesquiterpenes in different plants of the Myrtaceae family.

**Conclusions:**

The high quality chromosome-level rose myrtle genome and the comparative analysis of TPS gene family open new avenues for obtaining a higher commercial value of essential oils in medical plants.

**Supplementary Information:**

The online version contains supplementary material available at 10.1186/s12864-024-10509-6.

## Background

Volatile compounds play important roles in nature, such as altering plant-animal interactions and altering the local abiotic environment. The Myrtaceae species are regarded as essential oil producers because of the high concentration of cyclic mono- and sesquiterpenes [1–3]. Eucalyptus oil contains 1,8-cineole as the main component [4–6]. The leaves of *Melaleuca alternifolia* are dominantly detected with terpinen-4-ol, terpinolene, and 1,8-cineole, and these volatiles are usually called tea tree oil [7, 8]. Rose myrtle (*Rhodomyrtus tomentosa* (Ait.) Hassk), belonging to the family of Myrtaceae, is a paradigmatic example of terpenes-rich medicinal plant [9, 10]. Rose myrtle is increasingly used in a wide field of applications, including medicine, cosmetics, healthy food, and for industrial purposes [11, 12]. At present, many volatile compounds have been detected in rose myrtle [13], especially the leaves are enriched with (+)-α-pinene and β-caryophyllene [14, 15]. These simple and polymeric terpenoids function as photoprotectants, antifeedants, or physical barriers, playing vital roles in plant growth, development, and environmental interaction [16–18]. Commercially used essential oils, including myrtle oil, lavender oil and tea tree oil, are a mixture of volatile terpenes [19], with a growing amount of importance in industrial applications [20, 21].

Volatile terpenes are the largest class of natural products, which essentially originate from the C5 substrates dimethylallyl diphosphate (DMAPP) and isopentenyl diphosphate (IPP) [22]. The DMAPP and IPP precursors are produced through the mevalonate (MVA) and methylerythritol phosphate (MEP) pathways, respectively [23]. In plants, the MEP pathway typically operates in plastids while the MVA pathway operates in the cytosol [23]. Hemi-, mono-, and diterpenes, as well as carotenoids (tetraterpenes), are produced via the MEP pathway [24]. Terpene synthase (TPS) catalyzes complex carbocation cascade reactions on the prenyl diphosphate substrate, resulting in cyclic or linear terpene backbones [25, 26]. However, biology of volatile terpenes biosynthesis and accumulation is still unclear in rose myrtle.

*TPS* gene family members are divided into seven subgroups (-a, -b, -c, -d, -e/f, -g, and -h) [27, 28]. TPS-a and TPS-b mainly synthesizes sesquiterpenes and monoterpenes, respectively [27, 28]. TPS-g can synthesize monoterpenes, sesquiterpenes and diterpenes [27]. Terpenes and terpenoids play important roles in plant resistance to herbivores and response to environmental stimuli [29]. The genetic basis of terpene synthesis has been widely concerned in family Myrtaceae. The gene numbers encode putative terpene synthase in *Eucalyptus grandis*, *M. alternifolia*, and *Leptospermum scoparium* were 113, 37, and 49, respectively [7, 30, 31]. Further research into terpene biosynthesis is demanded for industrial production of essential oils.

A gap-free rose myrtle T2T genome has been reported recently [32], and their genome assembly provides a foundation for investigating the anthocyanin accumulation mechanism of *R. tomentosa*. However, as medicinal resources and undomesticated plants, the genetics of the special medicinal components and environmental adaptation strategies of *R. tomentosa* requires a better understanding and possible improvement. Here, we assembled a chromosome-level genome for *R. tomentosa* using third-generation PacBio in association with Illumina sequencing and Hi-C technique. Gene amplification and natural selection shaped the genetic adaptation of *R. tomentosa* to the harsh biotopes. The structure of the genes involved in the terpenoid synthesis pathway are positively selected. Our study represents the basis for exploring the genetic potential of *R. tomentosa* which contributes to the accumulation of essential oils.

## Materials and methods

### Plant sampling

For whole-genome assembly, a mature adult *R. tomentosa* individual was selected from a natural population from the South China National Botanical Garden (23.1817 N, 113.3671 E, Chinese Academy of Sciences, Guangzhou, China). The voucher specimen was kept at the South China Botanical Garden Herbarium (IBSC 0925721). Fresh leaves were collected for whole genome sequencing with Illumina HiSeq X Ten and PacBio Sequel sequencing platforms. For RNA sequencing in support of gene annotation, young leaves, petal lower lips, young stems, green fruits, and roots were sampled from the same individual.

### Genome sequencing, assembly and quality assessment

We extracted and purified the total DNA from fresh leaves. For Illumina short-reads sequencing, PCR-free libraries with 300bp, 500bp, and 10kb-20kb paired-end (PE) insert were prepared and sequenced on the Illumina HiSeq X Ten platform. SMRT long-read sequencing was performed on a PacBio Sequel platform with the Sequel Sequencing Kit 2.1. For Hi-C sequencing, young and fresh leaf tissues were preserved in 1% (vol/vol) formaldehyde, DNA was cross-linked according to protocol, and a single library (150-bp PE) was sequenced on the Illumina HiSeq X platform. More detailed information on sequencing can be found in Table S1 (see online supplementary material).

Wtdbg2 (v1.3.1) [33] and FALCON (v0.4.1) [34] were used for error correction in PacBio long reads according to Illumina short reads and then generate consensus sequences. Further, these subreads were assembled into contigs by Flye v0.2.1. We applied SSPACE v1.2.0 [35] to generate scaffolds using Illumina mate-paired reads. Preassembled scaffolds were clustered, ordered, and orientated onto pseudo-chromosomes with ALLHiC software (v0.8.11) [36]. The genome size was estimated based on k-mer distribution analysis by GenomeScope (v2.0) [37] using Illumina short reads without a flow cytometry analysis. Hi-C libraries of fresh young leaves were constructed with NEB Next Ultra II DNA library preparation kit and DpnII enzyme (Ipswich, MA, USA).

Benchmarking Universal Single-Copy Orthologs (BUSCO) v5.6.1 were used to evaluate the accuracy and completeness of the assembled genome. Genome completeness was assessed using the plant’s dataset of the BUSCO database, with an e-value < 1e-5. Single-copy embryophyta_odb10 homologous genes in BUSCO were used to predict the gene status of the existing sequences in the genome.

Finally, we used Merqury (v1.3) [38] to estimate the consensus QV of the assembly. Augustus [39] was utilized in de novo gene prediction while Trinity were implemented to generate EST evidence with RNA-seq data from four different tissues (root, leaf, flower, stem, and green fruit). The quality of assembled genome was evaluated by mapping RNA-seq reads from these different tissues using Bowtie2 [40].

### Chromosome counting and karyotype analysis

Root tips were pretreated with 0.002% hydroxyquinoline at 4 ℃ for 3 h [41]. After a thorough wash, tips were fixed in 1:3 acetic ethanol and digested in HCl (1 M) solution for 45 min in a 37 ℃ water bath. The root tips were stained with Carbol-fuchsin solution for 72 h, then cells were crushed onto a glass plate and drawn under oil immersionlens.

### Repeat and noncoding RNA annotation

We performed repeat masking using EDTA (v1.9.4 with parameter: –sensitive 1 –anno 1 –evaluate 1) with cDNA assembled from RNA-seq reads by Trinity. Four types of non-coding RNA genes, including tRNAs, rRNAs, miRNAs, and snRNAs, were predicted in the *R. tomentosa* genome. The tRNA genes were predicted using tRNAscan-SE with eukaryote parameters. INFERNAL with default parameters was used to annotate miRNA, snRNA, and rRNA.

### Structural and functional annotation of genes

A combined strategy of homology-based search, *de-novo* gene prediction, and RNA sequencing-aided annotation was used to annotate gene structure for the *R. tomentosa* genome. For homolog prediction, sequences of proteins from 13 species, including 6 closely related species from Myrtaceae (*E. grandis*, *L. scoparium*, *Psidium guajava*, *Syzygium oleosum*), other Myrtales species (*Punica granatum*, *Sonneratia alba*, *Rhizophora apiculata*, *Sonneratia caseolaris*), some representative species (*Arabidopsis thaliana*, *Solanum lycopersicum*, *Vitis vinifera*, *Vaccinium corymbosum*) and monocot species (*Oryza sativa*)*.* The protein sequences were aligned to the genome using tBlastn with an e-value cut-off of 1e-5. *De-novo* gene structure identification was based on Augustus [42], SNAP [43], and Fgensh, respectively. RNA-seq reads from different tissues were aligned to the genome using Bowtie2 (v3.2.7). Finally, putative protein-coding genes in the *R. tomentosa* genome were integrated using the Maker package (v 3.01.03).

Functional annotation of the protein-coding genes was conducted by performing BlastP (e-value cut-off 1e-05) searches against entries in the NCBI nr and SwissProt databases. Searches for gene motifs and domains were performed using InterProScan. The GO terms for genes were obtained from the corresponding InterPro or Pfam entry. Pathway reconstruction was performed using KOBAS (v2.0) and the KEGG database.

### Phylogenetic analysis and estimation of divergence time

OrthoFinder was used to identify orthologous genes from *R. tomentosa* and 13 other species including *A. thaliana*, *O. sativa*, *V. vinifera*, *E. grandis*, *L. scoparium*, *P. granatum*, *P. guajava*, *R. apiculata*, *S. alba*, *S. caseolaris*, *S. lycopersicum*, *S. oleosum*, and *V. corymbosum*. Single-copy orthologous genes were retrieved from these 14 species and aligned using MUSCLE [44] with default parameters and low-quality alignment regions were removed using Gblocks (v 0.91b) with default parameters. All alignments were combined to produce a super-alignment matrix, which was used to construct a maximum likelihood (ML) phylogenetic tree using RAxML (v8.2.12) with parameters: -f a ­- × 12,345 ­-p 12,345 ­-# 100 ­-m PROTGAMMALGX ­-s ex.fa.gb ­-n ex -T 30. Divergence times between species were calculated using the r8s with the default parameters.

### Gene family expansion and contraction analysis

Gene family expansion and contraction were conducted using the default settings by CAFÉ (v4.2.1) [45]. Gene families were identified by OrthoFinder. We determined the gene family expansions or contractions when the difference in gene copy number was significant with P-value < 0.01.

### Comparative genome analyses

To assess the degree of collinearity, we try to identify syntenic blocks among *R. tomentosa*, *P. guajava*, and *E. grandis* using MCScanX [46]. A syntenic region was highlighted if it contained at least 30 shared genes.

### Identification of *TPS* gene family

For the identification of *TPSs*, representative members of the subfamilies of *M. alternifolia*, *P. guajava*, and *R. tomentosa* were used as queries to perform Blastp searches against the protein database of each species with an *E*-value cut-off of 1e-5. Candidate sequences identified as orthologs were then aligned using Mafft to remove those that did not contain the intact domain. For phylogenetic analysis, sequences were combined to produce a super-alignment matrix, which was used to construct a maximum likelihood (ML) phylogenetic tree in RAxML (v8.2.12) with parameters: -f a ­- × 12,345 ­-p 12,345 ­-# 1000 ­-m PROTGAMMALGX ­-s ex.phy ­-n ex -T 30. The successfully constructed phylogenetic tree is displayed and annotated using iTOL software. Conserved motifs were identified by MEME tools, conserved domains were identified by NCBI Batch CD-search and visualized in TBtools-II [47].

### RNA extraction, library construction, and sequencing

Total RNA was extracted using a Trizol reagent kit (Invitrogen, Carlsbad, CA, USA) according to the manufacturer’s protocol. RNA quality was assessed on an Agilent 2100 Bioanalyzer (Agilent Technologies, Palo Alto, CA, USA) and checked using RNase-free agarose gel electrophoresis. The cDNA fragments were purified and ligated to Illumina sequencing adapters. The ligation products were size selected by agarose gel electrophoresis, PCR amplified, and sequenced using Illumina HiSeqTM 4000 with PE 150 bp. The unigene expression was calculated and normalized to TPM (transcripts per million).

### Positive selection analysis

For positive selection analysis, we first identified single-copy orthologous genes from *R. tomentosa* and the three most closely related species with assembled genomes: *L. scorparium* (Myrtaceae), *E. grandis* (Myrtaceae), and *P. guajava* (Myrtaceae), *S. oleosum* (Myrtaceae) and *P. granatum* (pomegranate, Lythraceae). For these genes, based on the phylogenetic topology, we employed the branch-site model incorporated in the PAML package v4.9 [48] to detect positively selected genes (PSGs). When one of the five species of Myrtaceae was specified as a foreground branch, the other four and the pomegranate branches in the phylogenetic tree were used as background branches. We conducted likelihood ratio tests to determine whether the positive selection was operating on the foreground branch. In this study, PSGs were identified only when P < 0.001.

## Results

### *De-novo* genome assembly and pseudo-chromosome construction

**Fig. 1:**
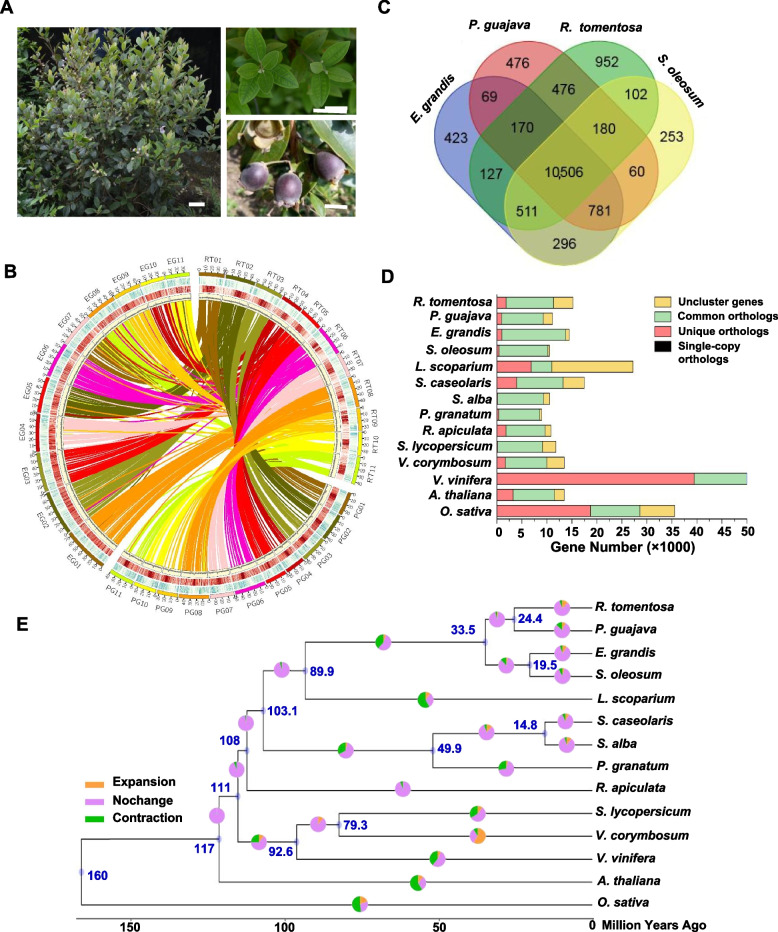
Plant morphology, genome features and phylogenetic relationships of *R. tomentosa*. **A** The phenotype of *R. tomentosa*. Bar = 2 cm; **B** Circos view of the *R. tomentosa* (RT), *P. guajava* (PG) and *E. grandis* (EG). From the outside to inside, Circle1: The assembled 11 chromosomes for three species; Circle2: Gene density plotted in a 50-kb sliding window; Circle3: Transposable element (TE) density plotted in a 50-kb sliding window; Circle4: GC content plotted in a 50-kb sliding window; Circle5: Genomic syntenic regions denoted by a single line represent a genomic syntenic region between *R. tomentosa* and *P. guajava*, *R. tomentosa* and *E. grandis*; **C** Venn diagram represents the shared and unique gene families in *R. tomentosa* with those in other species. Each number represents the number of gene families; **D** Summary of gene family clustering of *R. tomentosa* and 13 related species. Single-copy orthologs: 1-copy genes in ortholog group. Common orthologs: orthologs in all species. Unique orthologs: species-specific genes. Uncluster genes: genes not in any groups; **E** Phylogenetic relationship of *R. tomentosa* and other 13 plant species. The blue numerical value beside each node shows the estimated divergence time (MYA, million years ago). The pie chart shows the evolution of gene families, including expansion (orange), contraction (green), and no change (pink)

The genome of *R. tomentosa*, which is commonly grown in the South China including Guangzhou (Fig. 1A) was sequenced. The estimated genome size was 459 Mb based on the 31-mer depth distribution analysis of the sequenced short reads (Fig. S1). We obtained around 130 Gb of a high-quality dataset, including ~ 44.5 Gb of short-read sequences from three mate-pair libraries and ~ 85.8 Gb of Pacbio sequences, which represent ~ 300X coverage for the genome (Table S1). As shown in Fig. S2, the karyotype consists of 2n = 2X = 22 chromosomes. We assembled the genome with size of 466 Mb (Table 1, S2, and S3), consisting of 1,143 contigs with an N50 of 1.01 Mb (Table S2). Using Hi-C technology, 99.56% (463.9 Mb) of contigs can be ordered and anchored onto 11 pseudo-chromosomes (Fig. 1B, Table 1 and Fig. S3), which finally consisted of 28 scaffolds (11 pseudo-chromosomes and 17 scaffolds) with an N50 of 43.7 Mb (Table 1 and Table S2). The GC content of the assembled genome is 40.59% (Table 1), which is similar to those of *P. guajava* and *E. grandis*, the two closely related species to *R. tomentosa*.

**Table 1:** Summary statistics of the genome assembly and annotation of *R. tomentosa*

Feature	Value
Estimated genome size (Mb)	459
Total size of assembled scaffold (Mb)	466
N50 of scaffold (Mb)	43.7
Longest scaffold (Mb)	49.2
Number of scaffold	28
GC content (%)	40.59
Percentage of scaffolds archored in chromosome (%)	99.56
Number of contig	1,143
N50 of contig (Mb)	1.01
Percentage of repeat elements (%)	35.21
Number of gene models	35,554

The BUSCO database detected 1,546 (95.8%) and 24 (1.5%) complete and fragmented gene models, respectively out of 1,614 BUSCO genes (Table S3). To evaluate genome assembly quality, Merqury results showed that the integrity of the genome assembly was 86.2%, QV = 36.7, and the error rate was only 0.021%, indicating that a genome with high integrity and accuracy was constructed (Fig. S4). Moreover, 76.11%-95.50% of RNA-seq reads generated from different tissues can be successfully mapped to the assembled genome by hisat2 (Table S4). We also obtained the RNA-seq datasets of leaf samples reported by He et al. [14], and 75.33%-92.42% of RNA-seq reads were mapped to the assembled genome. Taken together, these observations suggest the high quality and completeness of the chromosome-level reference genome assembly of *R. tomentosa*.

### Repetitive elements and protein-coding gene annotation

Repeat sequence annotation showed that the *R. tomentosa* genome contained 35.21% of repetitive sequences (Table 1 and Table S5). Among these sequences, long terminal repeats (LTRs) were the most abundant interspersed repeats, occupying 32.06% of the genome, including 18% Gypsy LTRs and 5.33% Copia LTRs (Table S5). TIR repeats and helitron repeats accounted for 2.12% and 0.45%, respectively (Table S5). We confidently annotated 35,554 protein-coding genes of which 95.7% had a homolog in a suite of functional databases (Table 1 and Table S6). In addition, 2,892 noncoding RNAs, comprising 143 conserved microRNAs, 601 transfer RNAs, 1,754 ribosomal RNAs, and 394 small nuclear RNAs, were identified in the *R. tomentosa* genome (Table S7). These results indicated that a little higher number of genes were annotated in *R. tomentosa* compared with that of other species (Table S8). A comparison of gene models for *R. tomentosa* species revealed that the length of exons and intron in *R. tomentosa* was relatively conserved, whereas the length of introns is a little shorter in *A. thaliana* (Fig. S5). However, the average length of genes was a little shorter in *R. tomentosa*, compared with other species (Fig. S5).

### Gene family analysis

To identify evolutionary characteristics and gene families, the *R. tomentosa* genome was compared with 13 published genomes, including 6 closely related species from Myrtaceae (*E. grandis*, *L. scoparium*, *P. guajava*, *S. oleosum*), other Myrtales species (*P. granatum*, *S. alba*, *R. apiculata*, *S. caseolaris*), some representative species (*A. thaliana*, *S. lycopersicum*, *V. vinifera*, *V. corymbosum*), and a monocot rice (*O. sativa*) (Table S8). Based on gene family clustering analysis, 31,645 gene families were identified in total, of which 2,913 were shared by all 14 species, and 14 of these shared families were single-copy gene families (Table S9).

**Fig. 2:**
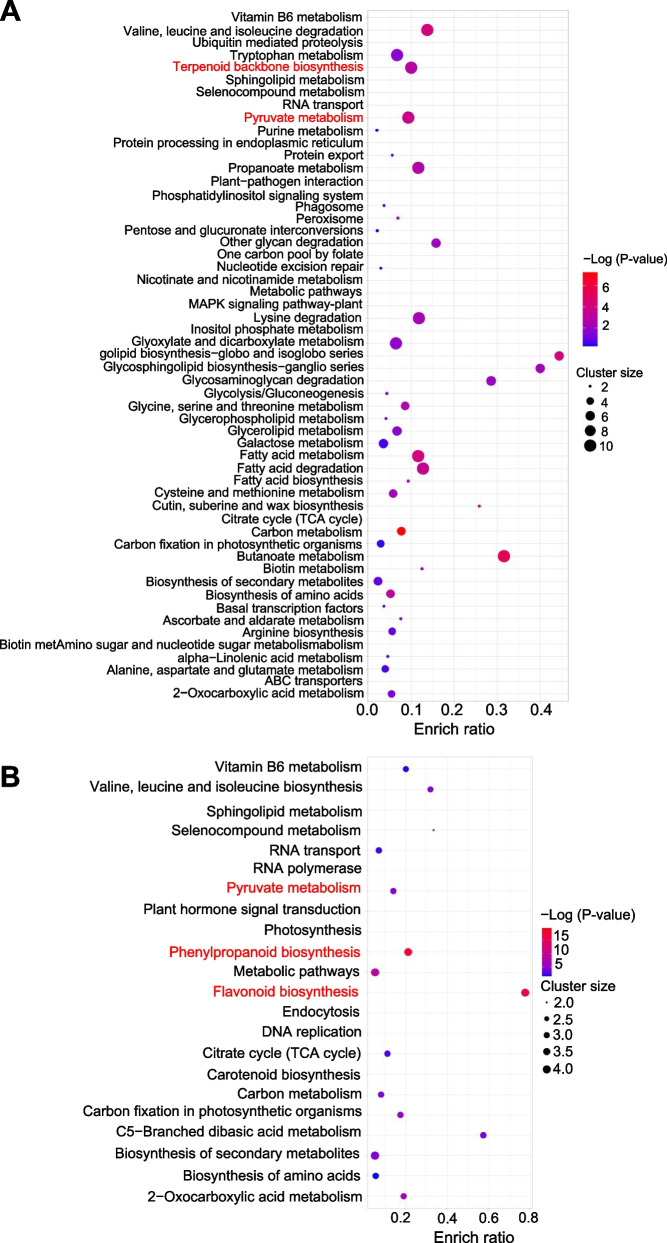
Gene expansion involved in terpenoid synthesis in *R. tomentosa*. **A** Enriched terms for specie-specific genes in *R. tomentosa*; **B** Enriched pathways for significant expansion genes in *R. tomentosa*

Gene family numbers were compared between *R. tomentosa* and other species. As shown in Fig. 1C, 10,506 gene families were shared between species, and 952 gene families were specific to *R. tomentosa*. Compared with *P. guajava*, there were more species-specific genes in *R. tomentosa* (Fig. 1D). Phylogenetic analysis of a concatenated sequence alignment of *R. tomentosa* and 13 other plant species indicated that *R. tomentosa*, as expected, clustered with Myrtaceae species (Fig. 1E). The divergence time between *R. tomentosa* and the most closely related species, *P. guajava*, was estimated to be ~ 24.4 million years ago (Fig. 1E). Gene Ontology (GO) and Kyoto Encyclopedia of Genes and Genomes (KEGG) enrichment analysis revealed that specific genes were especially enriched in terpenoid backbone biosynthesis and pyruvate metabolism (Table S10 and S11, Fig. 2A). Compared with the most recent common ancestor of the 14 plants, there were significant differences in the gene family in different species of Myrtaceae. In the case of the family gene in rose myrtle and eucalyptus, it tended to expand, while mainly experienced contraction in guava. Functional analysis showed that the significantly expanded genes were over-represented in ontology terms related to pyruvate metabolism, phenylpropanoid biosynthesis and flavonoid biosynthesis (Table S12 and S13, Fig. 2B). However, the contracted gene families did not show many specificities with marginal enrichment terms in phenylpropanoid biosynthesis (Table S14 and S15). These results suggested that gene expansion correlated with the terpenoid biosynthesis in *R. tomentosa*.

### Gene duplication affected terpenoids synthesis in *R. tomentosa*

**Fig. 3:**
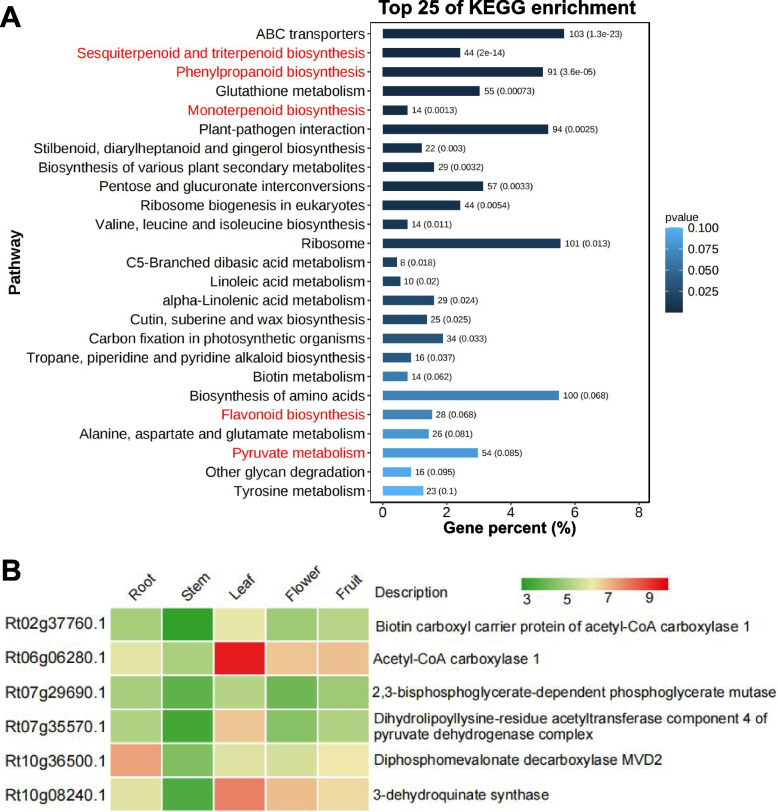
The adaptive evolution of *R. tomentosa* involved in terpenoid synthesis. **A** Top 25 of KEGG enrichment of duplicate genes in *R. tomentosa*; **B** Positively selected genes associated with terpenoid synthesis display tissue-differential expression under normal conditions in *R. tomentosa.* The color represents the gene expression values of TPM of genes transformed by log_2_

The distribution of synonymous substitutions per synonymous site (Ks) across all paralogous genes (regardless of gene order) showed a peak at Ks = 0.9, and similar peaks were found for *P. guajava* (Ks = 1.08) and *E. grandis* (Ks = 1.07) (Fig. S6). As shown in Fig. S6, the whole-genome duplication (WGD) event of *R. tomentosa* occurred later than that of *P. granatum*, which was consistent with previous publications in *R. tomentosa* [32]. These results provided additional evidence of one WGD event in Myrtaceae after the well-known paleo-hexaploidization event, γ, in the most recent common ancestor (MRCA) of all eudicots. We then analyzed the different origins of gene duplicates. All types of duplications were found and dispersed account for the largest proportion (32.4%), followed by the type of proximal (7.8%), tandem (5.4%) and WGD/segmental (0.7%) (Table S16)*.* Further analysis showed that the KEGG pathway was enriched in monoterpenoid biosynthesis, sesquiterpenoid and triterpenoid biosynthesis, pyruvate metabolism, flavonoid biosynthesis, and phenylpropanoid biosynthesis (Fig. 3A). These results provided clues about the potential contribution of the gene expansion and duplication on the accumulation of volatile substances.

### Positively selected genes (PSGs) in *R. tomentosa*

To better understand the evolutionary footprint in the accumulation of volatile compounds, we further analyzed the positive selection genes in *R. tomentosa*. Positive selection analyses for *R. tomentosa*, *P. guava*, *E. grandis*, and *E. citriodora* were conducted using the orthologs from *P. granatum* as the outgroup. We identified 872 genes possibly under positive selection in *R. tomentosa* among the 3,923 single-copy orthologous genes (*P* < 0.001; Table S17). A GO functional classification of PSGs indicated that the terms associated with DNA repair, protein ligase, membrane-bounded organelle, intracellular membrane-bounded organelle, and vesicle transport were significantly over-represented (Fig. S7). We found six PSGs involved in terpenoid synthesis (Table S17). Moreover, these PSGs were detectable at the transcriptional level in various stages of development, especially in leaf (Fig. 3B, Table 2). We also identified three positive selection genes related to the stomatal development pathway (Table S17). Synthetically, these results indicated that *R. tomentosa* exhibited a remarkable pattern of adaptive evolution in response to environmental cues.

**Table 2:** Positive selected genes associated with terpenoid synthesis in *R. tomentosa*

Gene ID	m1	m2	2Δ	χ2 test	Description
Rt02g37760.1	-1837.14109	-1877.2746	80.267	3.27E-19	Biotin carboxyl carrier protein of acetyl-CoA carboxylase 1, chloroplastic
Rt06g06280.1	-12,104.3397	-12,114.0748	19.47	1.02E-05	Acetyl-CoA carboxylase 1
Rt07g29690.1	-1833.57896	-1843.00095	18.844	1.42E-05	2,3-bisphosphoglycerate-dependent phosphoglycerate mutase
Rt07g35570.1	-2947.5384	-2983.89484	72.713	1.5E-17	Dihydrolipoyllysine-residue acetyltransferase component 4 of pyruvate dehydrogenase complex, chloroplastic
Rt10g08240.1	-2810.46236	-2840.63792	60.351	7.94E-15	3-dehydroquinate synthase, chloroplastic
Rt10g36500.1	-2378.02701	-2419.20651	82.359	1.13E-19	Diphosphomevalonate decarboxylase MVD2, peroxisomal
Rt10g08240.1	-2810.46236	-2840.63792	60.351	7.94E-15	3-dehydroquinate synthase, chloroplastic

### *TPS* family genes probably affect terpenoids synthesis

**Fig. 4:**
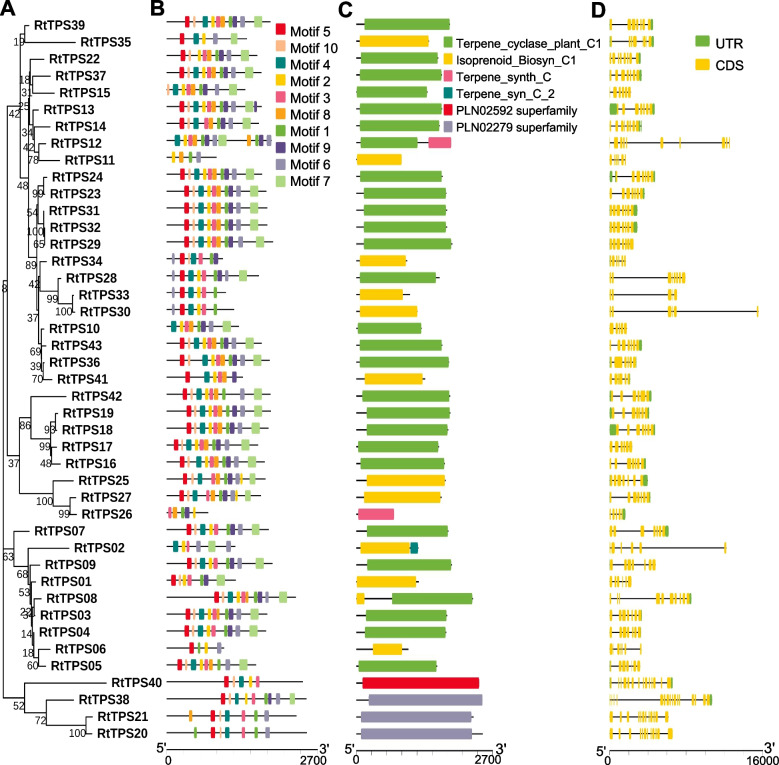
Analysis of phylogenetic tree, conserved motif, conserved domain and gene structure of *TPS* gene family in *R. tomentosa.* **A** Phylogenetic tree analysis of *TPS* genes in *R. tomentosa*; **B** Conserved motifs identified by MEME tools and visualized in TBtools; **C** Conserved domain of *RtTPSs*; **D** *TPS* genes structure of *R. tomentosa*

To infer the influence of the *TPS* family on terpenoid biosynthesis in *R. tomentosa*, molecular evolutionary analysis was conducted. In total, 43 and 32 *TPS* genes were identified in *R. tomentosa* and *P. guava* (Table 3, Table S18), respectively, which contained 7 previously reported *RtTPS* genes [14]. To gain further insights into the *RtTPS* gene members, we surveyed the evolutionary relationships (Fig. 4A), motifs (Fig. 4B), domains (Fig. 4C), gene structure and chromosomal location of each *TPS* gene copy (Fig. 4D, Fig. S8). The *RtTPS* genes were classified into six subgroups based on their conserved domain structures. Gene structure and conserved domain analysis revealed that all TPS had conserved domain associated with terpene biosynthesis (Fig. 4), which suggests a conserved function in these *RtTPSs*. These results revealed both conservation and divergence between each subfamily in *RtTPSs*.

**Table 3:** Experession levels of structural genes associated with terpenoid synthesis in *R. tomentosa*

Gene ID	Gene Name	Root	Stem	Leaf	Flower	Green Fruit
Rt01g34820.1	RtTPS01	0.00	0.00	0.00	0.00	0.00
Rt01g35150.1	RtTPS02	0.00	0.00	0.00	0.00	0.00
Rt01g35190.1	RtTPS03	0.00	0.00	9.45	0.00	0.00
Rt01g35240.1	RtTPS04	0.00	0.00	0.00	0.00	0.00
Rt01g35280.1	RtTPS05					
Rt01g35340.1	RtTPS06	0.39	0.00	1.68	0.30	1.48
Rt01g36570.1	RtTPS07	5.72	2.31	0.44	0.14	1.38
Rt01g36600.1	RtTPS08	1.60	0.00	3.59	3.15	0.08
Rt01g36880.1	RtTPS09	0.40	2.64	4.33	2.99	0.20
Rt02g32330.1	RtTPS10	0.00	2.04	0.43	0.00	0.00
Rt03g18180.1	RtTPS11					
Rt03g18580.1	RtTPS12	0.00	0.00	0.00	0.00	0.00
Rt03g18650.1	RtTPS13	0.00	2.48	0.11	0.00	1.57
Rt03g18780.1	RtTPS14	0.00	0.00	0.00	0.00	0.00
Rt03g31640.1	RtTPS15	0.00	0.00	0.00	0.00	0.00
Rt04g05070.1	RtTPS16	0.00	2.96	3.60	0.00	0.12
Rt04g05100.1	RtTPS17	0.00	1.55	0.00	0.00	0.00
Rt04g05150.1	RtTPS18	0.00	0.75	4.90	0.00	2.73
Rt04g05210.1	RtTPS19	0.00	0.97	0.92	0.00	0.00
Rt04g06670.1	RtTPS20	0.00	0.12	0.96	0.00	0.00
Rt04g07360.1	RtTPS21	0.00	0.00	0.00	0.00	0.00
Rt04g21190.1	RtTPS22	0.00	3.39	0.91	0.00	4.10
Rt04g22450.1	RtTPS23	0.00	0.00	1.93	0.00	0.00
Rt04g22530.1	RtTPS24	0.00	0.00	0.00	0.00	5.25
Rt05g07930.1	RtTPS25	0.00	0.00	2.84	0.00	0.00
Rt05g07940.1	RtTPS26	0.00	0.00	0.00	0.00	0.00
Rt05g07970.1	RtTPS27	0.00	1.39	1.82	1.22	0.36
Rt05g46070.1	RtTPS28	0.00	0.00	0.00	0.00	0.00
Rt05g46080.1	RtTPS29	0.00	1.96	3.75	0.07	0.00
Rt05g46260.1	RtTPS30	0.00	1.04	0.00	0.00	0.00
Rt05g46270.1	RtTPS31	0.00	4.50	4.75	0.35	0.00
Rt05g46310.1	RtTPS32	0.00	4.20	5.68	1.00	0.00
Rt05g46320.1	RtTPS33	0.00	1.57	0.40	0.00	0.00
Rt05g46350.1	RtTPS34	0.00	0.00	0.00	0.00	0.00
Rt06g55760.1	RtTPS35	0.00	0.00	0.00	0.00	0.00
Rt06g55780.1	RtTPS36	0.00	2.47	5.53	0.00	0.61
Rt06g55910.1	RtTPS37	0.00	2.07	0.00	5.81	0.00
Rt06g61740.1	RtTPS38	3.87	3.95	3.44	2.77	3.94
Rt07g14400.1	RtTPS39	0.00	0.00	7.50	0.00	3.35
Rt08g54350.1	RtTPS40	0.00	0.43	0.00	0.00	0.01
Rt09g45380.1	RtTPS41	0.00	0.00	0.00	0.00	0.00
Rt11g10340.1	RtTPS42	0.00	0.00	2.83	0.00	4.35
Rt11g48770.1	RtTPS43	0.00	2.86	5.17	3.57	5.24
Rt04g01780.1	RtAACT1	6.58	3.01	5.14	4.95	5.38
Rt08g59010.1	RtAACT2	8.49	3.82	7.11	6.49	8.07
Rt04g37120.1	RtHMGS1	7.55	4.58	6.35	6.15	6.69
Rt05g44800.1	RtHMGS2	3.99	3.62	5.33	4.36	3.34
Rt02g31690.1	RtMVK1	6.32	2.74	4.79	5.12	6.06
Rt10g36500.1	RtMVD1	7.45	4.50	6.05	5.89	6.40
Rt05g37040.1	RtPMK1	6.14	2.24	5.40	5.06	5.28
Rt03g38790.1	RtDXS1	3.99	2.95	4.27	5.63	4.51
Rt03g55960.1	RtDXS2	0.00	2.74	0.00	0.00	0.00
Rt03g55980.1	RtDXS3	0.00	1.45	0.00	0.00	0.00
Rt03g55990.1	RtDXS4	0.00	1.80	0.00	0.00	0.00
Rt08g20770.1	RtDXS5	5.36	4.86	6.66	3.84	5.84
Rt09g09640.1	RtDXS6	2.39	1.51	2.94	1.88	0.36
Rt09g15370.1	RtMCT1	2.84	3.51	4.87	2.82	3.52
Rt05g43430.1	RtMCS1	6.35	2.26	6.74	6.01	6.22
Rt09g42160.1	RtHDR1	6.59	3.61	8.25	7.61	7.55
Rt02g05320.1	RtHDS1	5.39	1.92	7.13	7.75	7.24
Rt01g36150.1	RtIPPI1	7.56	4.62	6.46	4.81	6.55
Rt01g36180.1	RtIPPI2	1.17	2.75	1.67	0.60	1.76
Rt01g36300.1	RtIPPI3	0.00	0.00	0.00	0.00	0.00
Rt01g36350.1	RtIPPI4	1.15	1.37	3.17	1.91	4.26
Rt08g53750.1	RtIPPI5	5.74	2.42	6.67	5.46	6.37

**Fig. 5:**
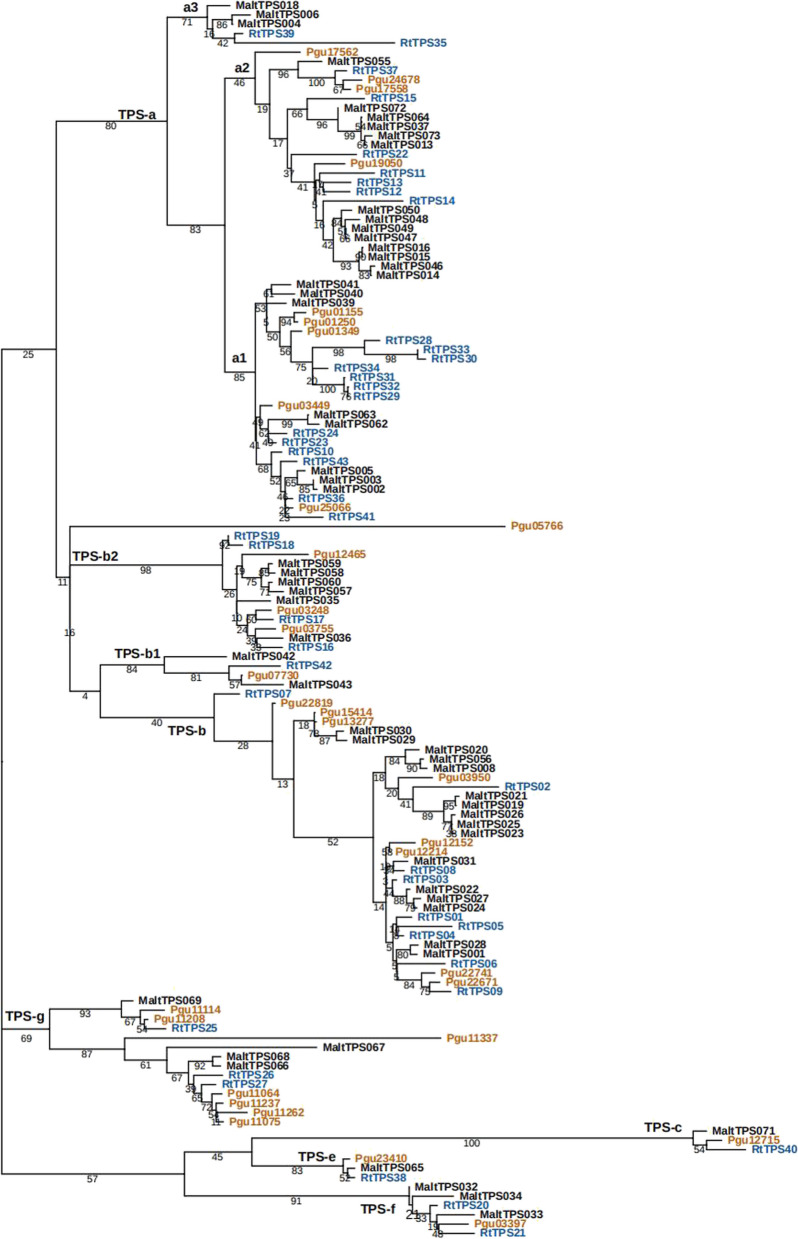
Evolutionary analysis of *RtTPS* genes. Phylogenetic tree analysis of *TPS* genes in *M. alternifolia*, *P. guava*, and *R. tomentosa* using the maximum likelihood method in RAxML with the bootstrap test (1,000 replicates) and annotated using iTOL software

In order to get a better understanding of the evolutionary relationship and classification of the *RtTPS* members, a ML phylogenetic tree was generated based on amino acid sequences of the TPS domains from *M. alternifolia*, *P. guajava*, and *R. tomentosa* (Fig. 5). Compared with *P. guava*, we found a specifically expanded TPS-a subgroup in *M. alternifolia*, and *R. tomentosa*, which may be related to the accumulation of sesquiterpenoid compounds, such as β-caryophyllene. In TPS-a subgroup, we found that the a1 subgroup was enriched with *RtTPSs*, but *MaltTPS* were predominantly clustered to a2 subgroup. Additionally, TPS-a3 clade was missing completely in *P. guava*. It suggested that the diversity of TPS-a subgroups led to the accumulation of special sesquiterpenes in different plants of Myrtaceae family. Compared *R. tomentosa* and *M. alternifolia*, TPS-g subgroup was enriched with *TPS* family genes of *P. guava*.

**Fig. 6:**
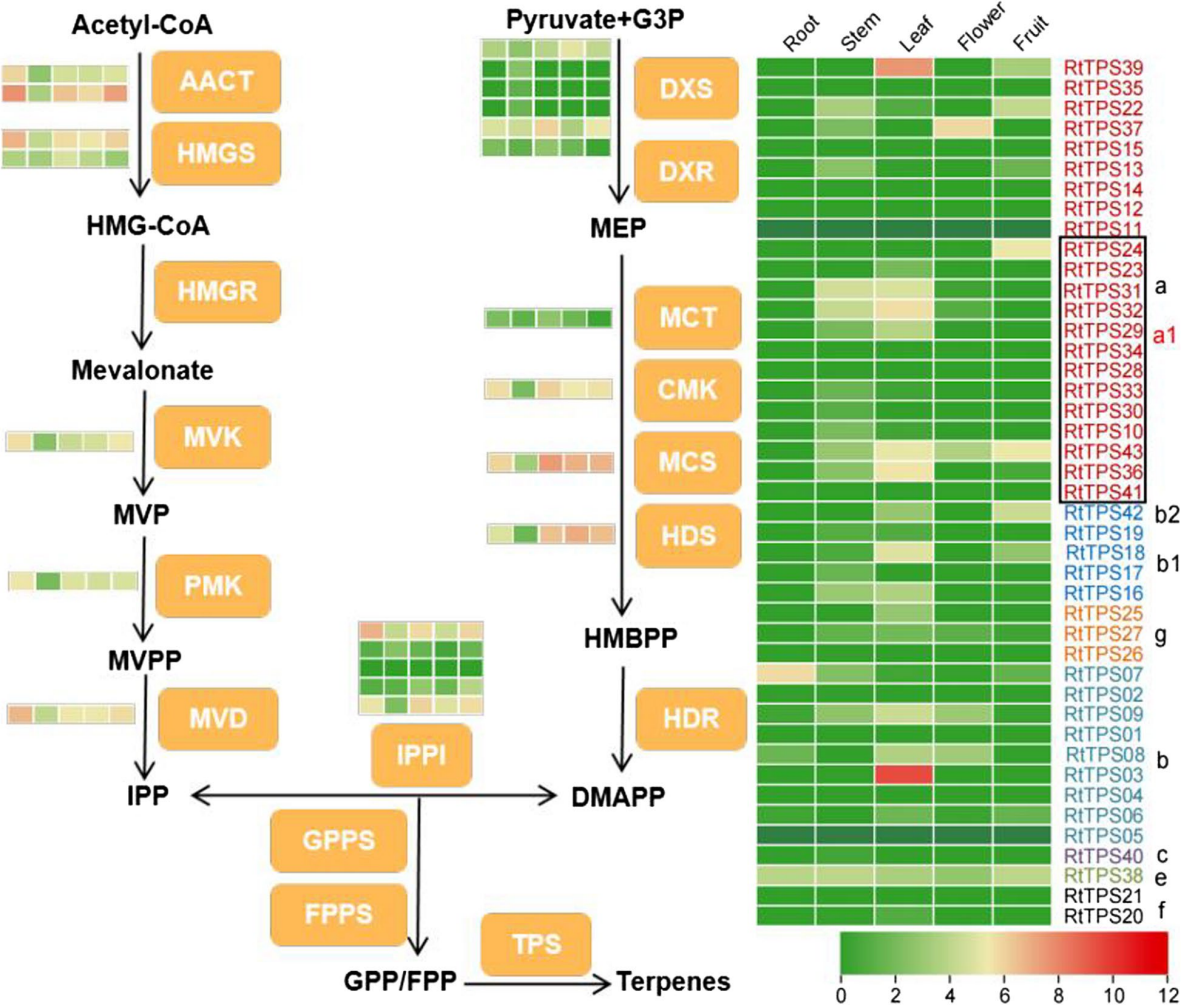
Expression profiles of *TPSs* in different tissues, i.e., root, stem, leaf, flower, green fruit, yellow fruit, and red fruit by RNA sequencing. HMG-CoA, 3-hydroxy-3-methyl glutaryl coenzyme A; MVP, mevalonate 5-phosphate; MVPP, mevalonate 5-diphosphate; IPP, isopentyl diphosphate; G3P, D-glyceraldehyde 3-phosphate; MEP, 2-C-methyl-D-erythritol 4-phosphate; HMBPP, 1-Hydroxy-2-methyl-2-butenyl 4-diphosphate; DMAPP, Dimethylallyl diphosphate; GPP, geranyldiphosphate; FPP, farnesyldiphosphate; AACT, acetyl-CoA-acetyltransferase; HMGS, hydroxymethylglutaryl-CoA synthase; HMGR, hydroxymethylglutaryl-CoA reductase; MVK, mevalonate kinase; PMK, phosphomevalonate kinase; MVD, diphosphomevalonate decarboxylase; DXS, 1-deoxy-D-xylulose-5-phosphate synthase; DXR, 1-deoxy-D-xylulose-5-phosphate reductoisomerase; MCT, 2-C-methyl-D-erythritol 4-phosphate cytidylyltransferase; CMK, 4-diphosphocytidyl-2-C-methyl-D-erythritol kinase; MCS, 2-C-methyl-D-erythritol 2,4-cyclodiphosphate synthase; HDS, (E)-4-hydroxy-3-methylbut-2-enyl-diphosphate synthase; HDR, 4-hydroxy-3-methylbut-2-enyldiphosphate Reductase; IPPI, isopentenyl-diphosphatedelta-isomerase; GPPS, geranyldiphosphate synthase; FPPS, farnesyldiphosphate synthase. Heatmap shows the transcript expression (log_2_TPM) across all samples. Color codes: red–higher expression; green–lower expression. From left to right the heatmaps represent different tissues (root, stem, leaf, flower, and fruit)

To probe the underlying mechanism of the terpene accumulation pattern, we drawn a predicted terpene biosynthesis pathway with the expression of structural genes in different tissues of *R. tomentosa* using transcriptome data (Fig. 6). Different structural genes participating in the cytosolic MVA pathway and plastid MEP pathway were identified in this study, exhibiting distinct expression patterns. Tissue-specific expression analysis revealed that the *RtTPS* genes were differentially expressed in various rose myrtle tissues. *RtTPS* family genes were dominantly increased in leaf and with a low expression in root, especially in TPS-a1 subgroup. Additionally, we found that two *TPS* genes, *RtTPS03* and *RtTPS39*, belonging to TPS-a and -b subgroups, were highly expressed in leaf. The results showed that *RtTPS* family genes affected characteristic terpene accumulation by specifically expanded subgroup and functional differentiation.

## Discussion

### Diversity among genome datasets contributes to comparative genomics analysis

Rose myrtle belongs to the family Myrtaceae [49], which has attracted increased attention recently because of its industrial and economic applications. A gap-free rose myrtle T2T genome has been reported recently during the period when we prepared the manuscript [32]. The genome size, GC contents, genome structure, and gene numbers of the *R. tomentosa* genome presented here is quite similar to the reported gap-free genome. These results indicated our assembly was of high quality, and it will provide useful datasets for comparative genomics. Another genome is subsequently reported, but the genome size (442 Mb) is smaller than the gap-free genome and our genome [50].

### Tandem duplication and specific subfamily expansion of *TPS* in *R. tomentosa*

This manual annotation of the rose myrtle genome revealed that genes and pseudogenes from the same *TPS* subfamily with high sequence similarities were frequently located in close proximity on the same chromosome. This marked clustering of *TPS* genes into tandem arrays in rose myrtle paralleled the tandem clusters found in *M. alternifolia* [51]. Our study found that the gene duplication through unequal crossing over, and subsequent sub- or neo-functionalization, or the expanded specific subfamily evolution were critical mechanisms underpinning the evolution of *TPS* in rose myrtle. The mechanisms of tandem duplication and specific subfamily expansion are considered as contributors to the adaptive diversification of genes [52], such as TPS family genes, as they are more likely to be retained following gene duplication due to stress pressures [53].

### The distinct gene subgroups of *TPS* affect specific terpene accumulation

All angiosperm *TPS* subfamilies are represented in *R. tomentosa* but variations in the size of certain subfamilies relative to the other Myrtaceae were observed [2].The largest distinction were evident in subfamilies that produce secondary metabolites, and thus are likely to be subject of adaptive pressures. For example, *R. tomentosa* has twice as many TPS-a (sesquiterpenoid) genes compared to *P. guava*, which is similarity to *M. alternifolia* [51]. This subgroup in rose myrtle is likely to have had the same significance historically as it had in *M. alternifolia*, which contributed to the abundance of aromatic compounds. *P. guava* has more TPS-g subgroup genes than *M. alternifolia* and *R. tomentosa*, and these results indicate that the distinctive gene subgroups of *TPS* led to the biosynthesis and accumulation of different aromas. We particularly find that TPS-a1 subgroup genes were significantly expanded and thus are key potential targets to produce β-caryophyllene in *R. tomentosa* [14]. The aromatic compounds and essential oils present are a key indicator in determining the economic value of *R. tomentosa* [54]*.* Then an in-depth understanding of terpene metabolism will help improve the potential application of secondary metabolites.

## Conclusion

We presented a high-quality chromosome-level reference genome for *R. tomentosa*. The genome characterization including the genome size, GC content, genome structure, gene number, duplication of the genome and divergent time with the close relatives were quite consistent with a recently reported gap-free *R. tomentosa* genome. Elaborate genomic information on *R. tomentosa* has primely illustrated the evolutionary relationship of *TPS* gene family associated with terpene accumulation, especially the TPS-a subfamily which plays an important role in synthesizing the special terpene. Our study provides a further opportunity to research the potential application of secondary metabolites among Myrtaceae in the future.

### Supplementary Information

Supplementary Material 1: Fig S1. K-mer frequency distribution curve of Illumina short reads for the *R. tomentosa* genome by GenomeScope. Fig S2. Chromosome karyotype analysis of *R. tomentosa*. 2n = 2X = 22. Bar = 10 μM. Fig S3. Hi-C contact data mapped to the *R. tomentosa* chromosome. Fig S4. Merqury assembly spectrum plots for evaluating k-mer completeness to the *R. tomentosa* chromosome. Fig S5. Comparison of gene models between *R. tomentosa* with those in other species. Fig S6. Distribution of synonymous substitution levels (Ks) of paralogous (A) and orthologous genes (B). Fig S7. Go analysis of positively selected genes in *R. tomentosa*. Fig S8. Chromosomal location of *TPSs* on chromosomes in *R. tomentosa*.

Supplementary Material 2: Table S1. Summary statistic for raw sequencing dataset. Table S2. Summary statistics for the final genome assembly of *R. tomentosa*. Table S3. Evaluation of the genome assembly of *R. tomentosa* using Benchmarking Universal Single-Copy Orthologs (BUSCO). Table S4. Statistics of the *R. tomentosa* RNA-Seq data from different tissues. Table S5. Statistics of the repeat annotation results. Table S6. Statistics of gene annotation. Table S7. Statistics of non-coding gene annotation. Table S8. List of plant genome sequences used in the comparative genomic analysis. Table S9. Gene families clustered by OrthoFinder in 14 species. Genes used for OrthoFinder were proteins without splice variants. Table S10. KEGG enrichment analysis of species-specific genes in *R. tomentosa*. Table S11. GO enrichment analysis of species-specific genes in *R. tomentosa*. Table S12. KEGG enrichment analysis of significant expansion genes in *R. tomentosa*. Table S13. GO enrichment analysis of significant expansion genes in *R. tomentosa.* Table S14. KEGG enrichment analysis of significant contraction genes in *R. tomentosa*. Table S15. GO enrichment analysis of significant contraction genes in *R. tomentosa*. Table S16. Classification of different origins of duplicate genes in *R. tomentosa*. Table S17. Positive selection genes in *R. tomentosa.* Table S18. The *RtTPS* genes in *R.tomentosa.*.

## Data Availability

The *R. tomentosa* genome assembly and transcriptome raw reads were submitted to BIG Sub (https://ngdc.cncb.ac.cn/search/specific?db=bioproject&q=PRJCA013967).

## Abbreviations

DMAPP: Dimethylallyl Diphosphate
IPP: Isopentenyl diphosphate
MVA: Mevalonate
MEP: Methylerythritol phosphate
TPS: Terpene synthase
BUSCO: Benchmarking Universal Single-Copy Orthologs
TPM: Transcripts per million
PSGs: Positively selected genes
KEGG: Kyoto Encyclopedia of Genes and Genomes
WGD: Whole-genome duplication
MRCA: Most recent common ancestor
