# Supplementary material for: Genomic analysis based on chromosome-level genome assembly reveals Myrtaceae evolution and terpene biosynthesis of rose myrtle
Source: BMC Genomics. 2024 Jun 10;25:578. doi: 10.1186/s12864-024-10509-6 (PMC11165866; doi:10.1186/s12864-024-10509-6)

Fig S1. K-mer frequency distribution curve of Illumina short reads for the *R. tomentosa* genome by GenomeScope.

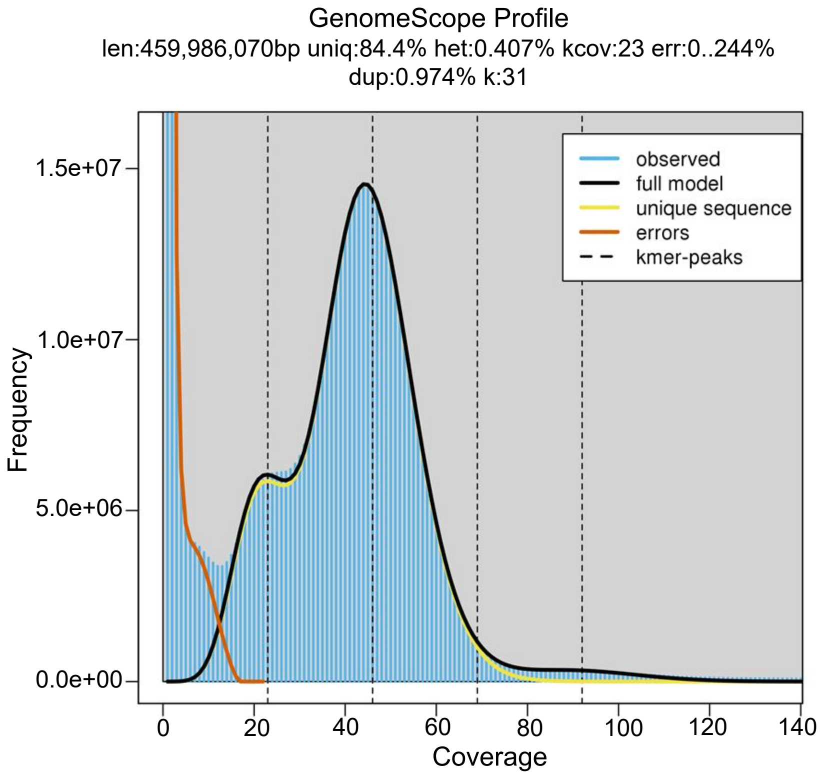

Fig S2. Chromosome karyotype analysis of *R. tomentosa*. 2n=2X=22. Bar=10 μM.

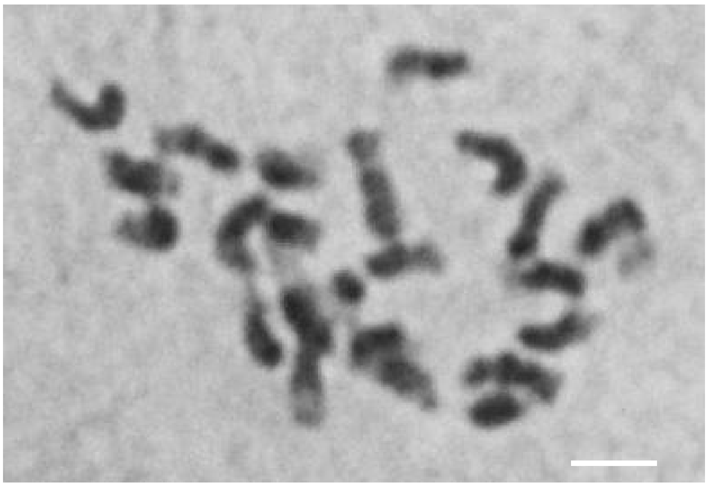

Fig S3. Hi-C contact data mapped to the *R. tomentosa* chromosome.

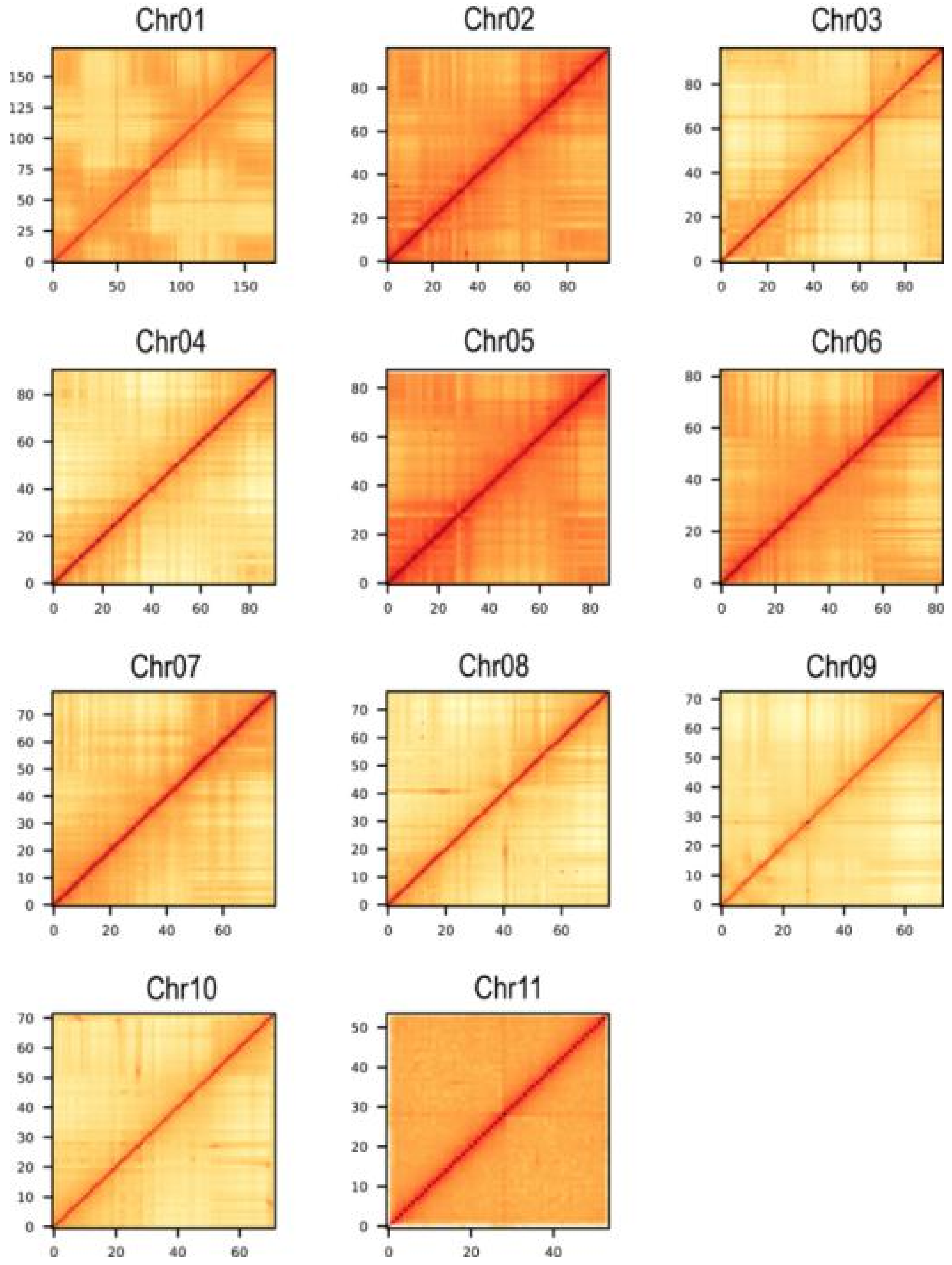

Fig S4. Merqury assembly spectrum plots for evaluating k-mer completeness to the *R. tomentosa* chromosome.

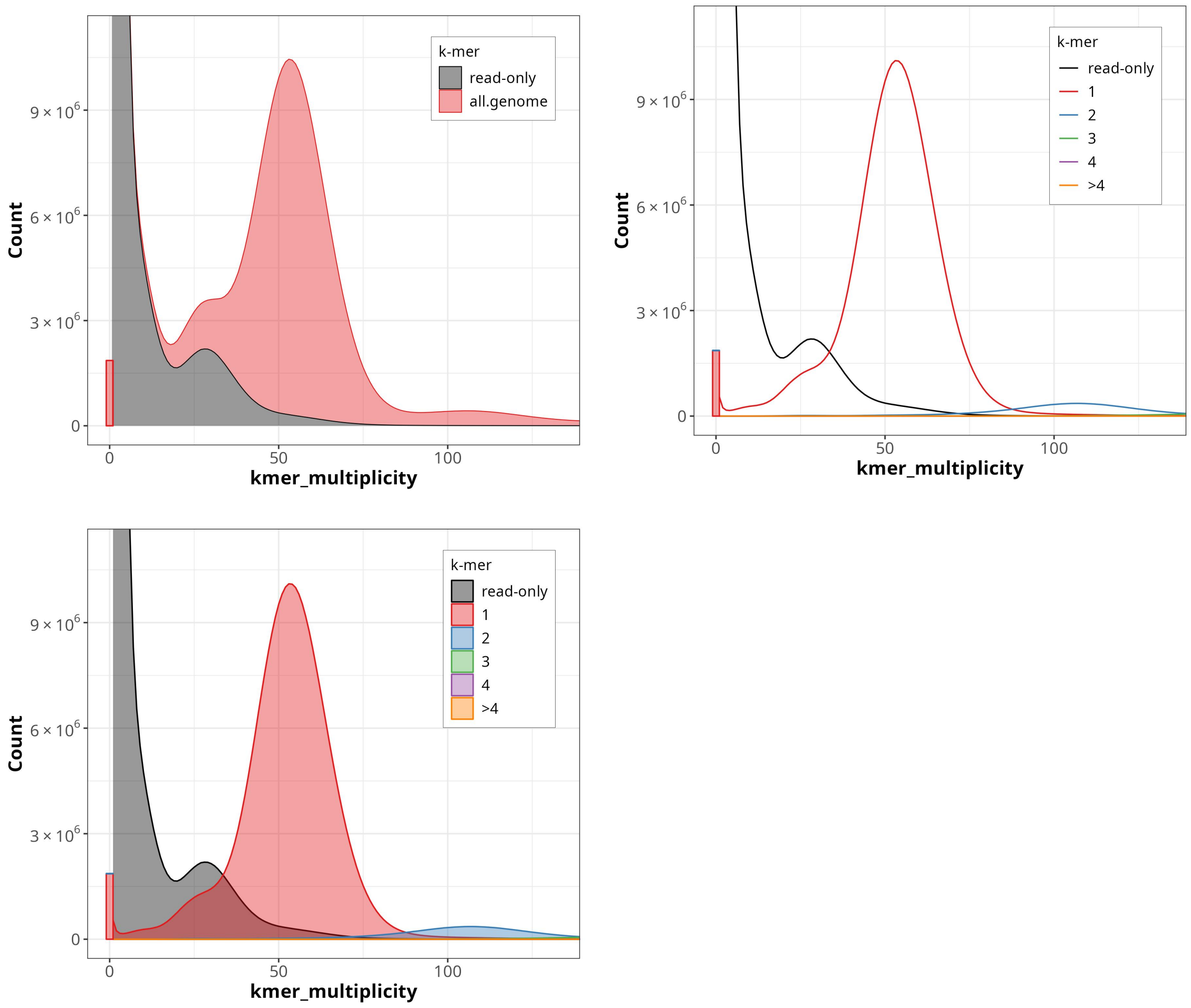

Fig S5. Comparison of gene models between *R. tomentosa* and other species.

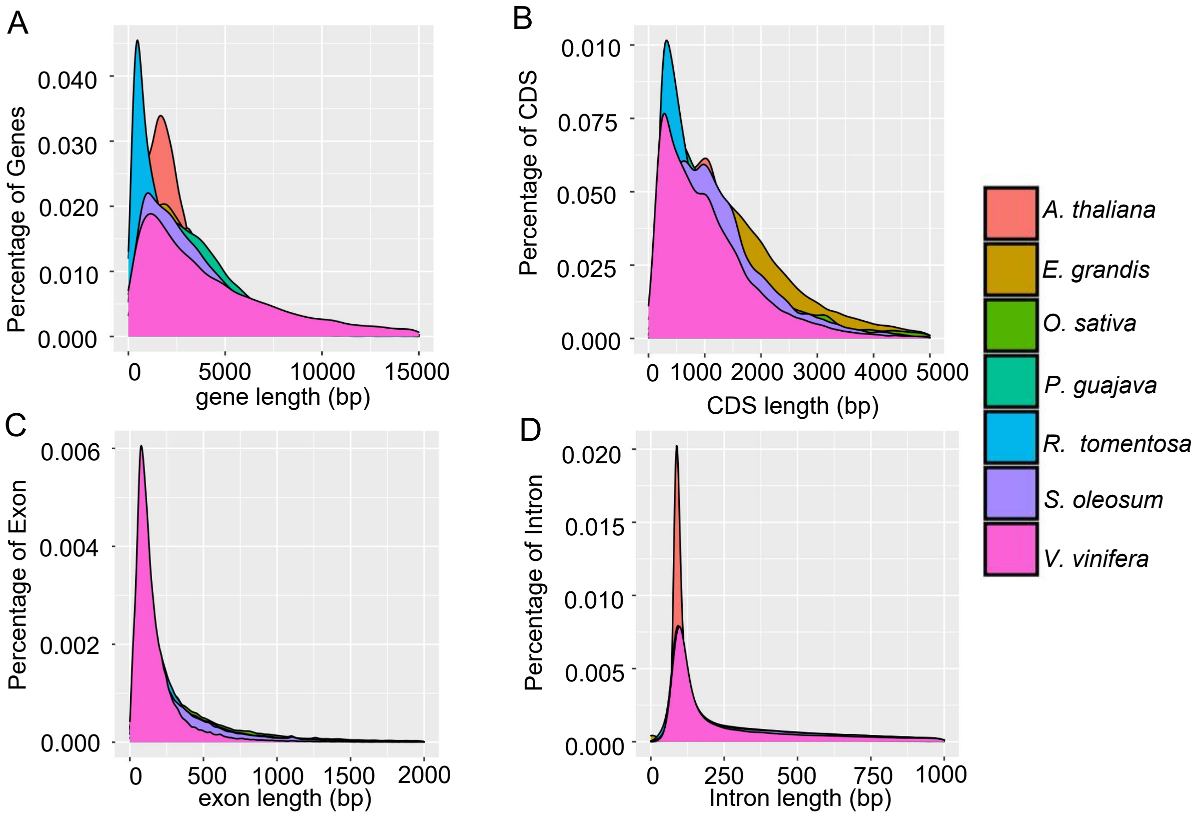

Fig S6. Distribution of synonymous substitution levels (Ks) of paralogous (A) and orthologous genes (B).

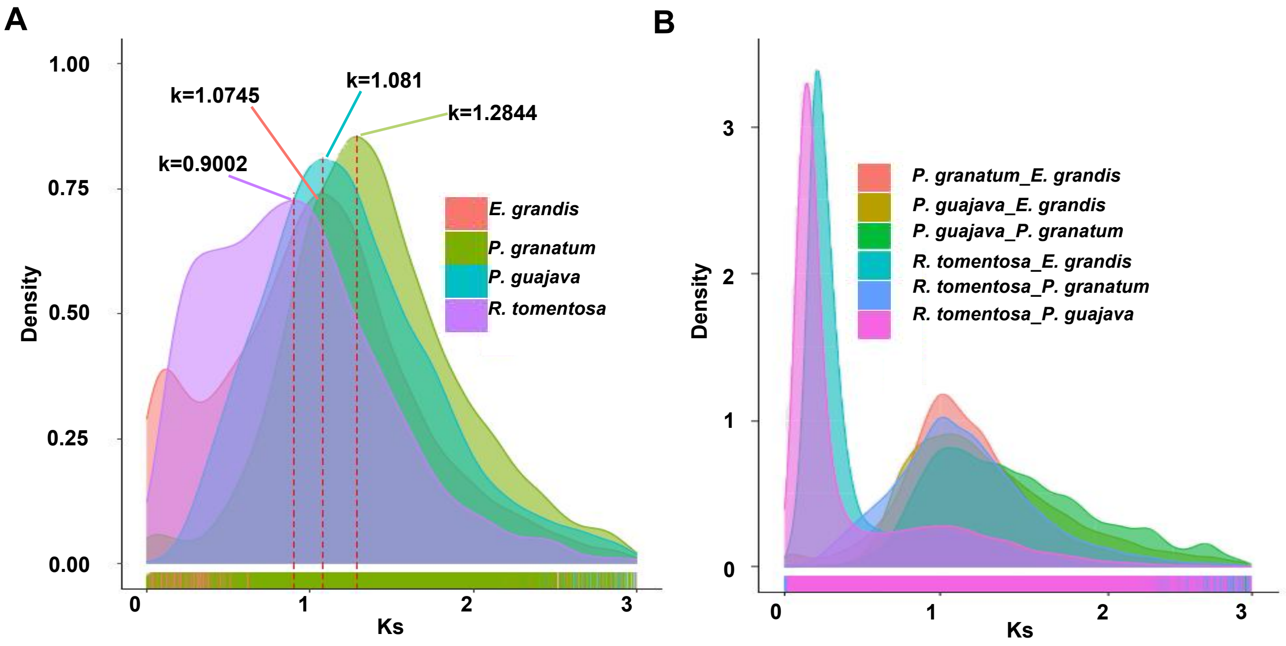

Fig S7. GO analysis of positively selected genes in *R. tomentosa*.

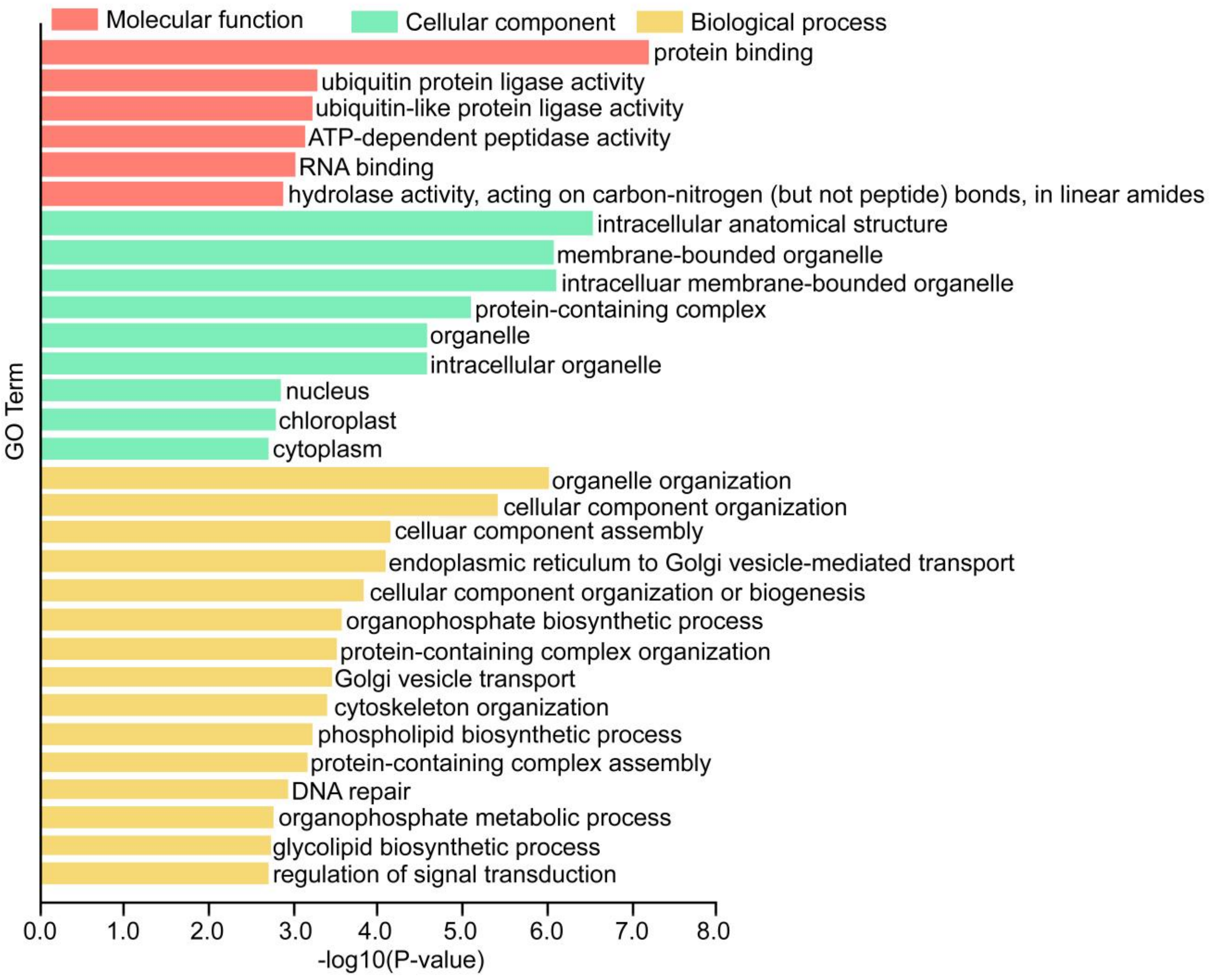

Fig S8. Chromosomal location of *TPSs* on chromosomes in *R. tomentosa*.

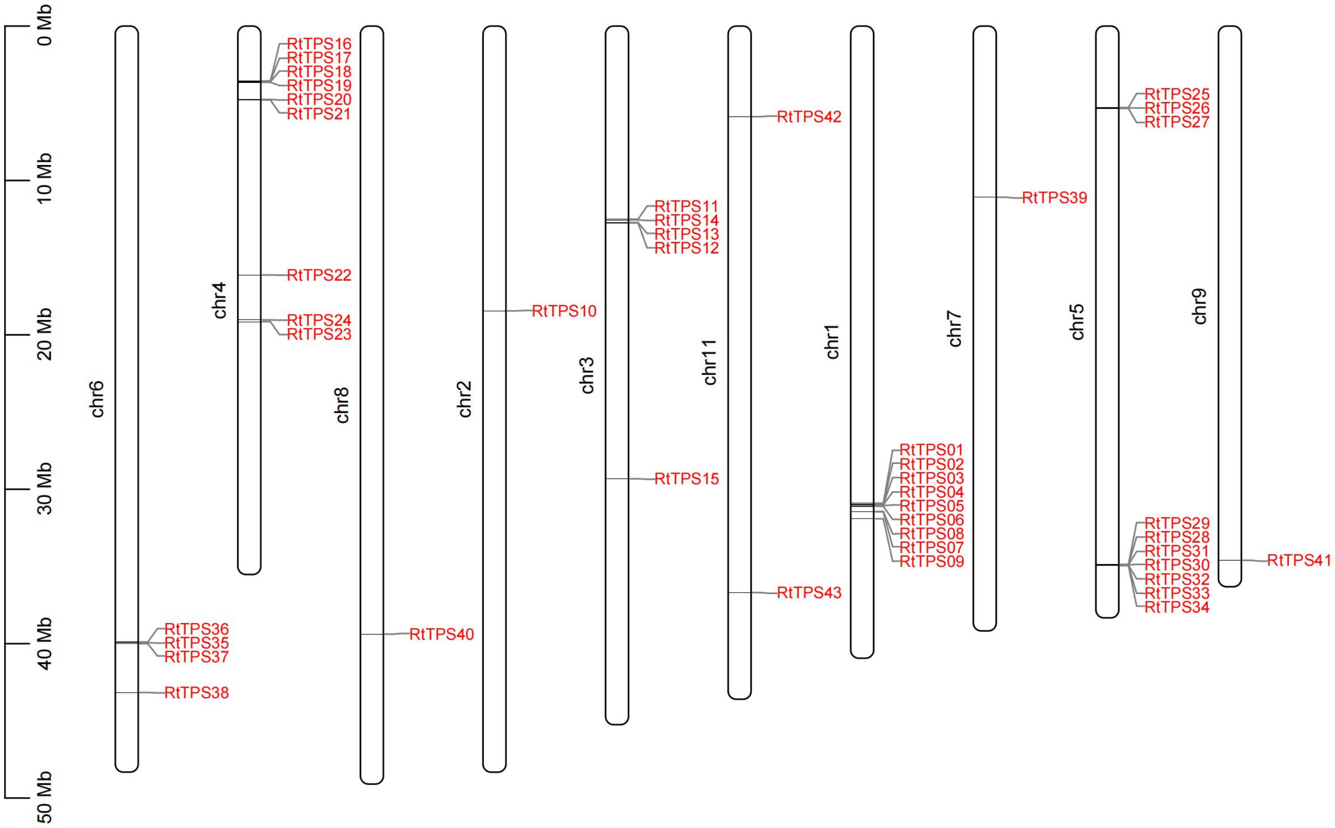

Supplement: Supplementary file 1 — Supplementary Material 1: Fig S1. K-mer frequency distribution curve of Illumina short reads for the R. tomentosa genome by GenomeScope. Fig S2. Chromosome karyotype analysis of R. tomentosa. 2n = 2X = 22. Bar = 10 μM. Fig S3. Hi-C contact data mapped to the R. tomentosa chromosome. Fig S4. Merqury assembly spectrum plots for evaluating k-mer completeness to the R. tomentosa chromosome. Fig S5. Comparison of gene models between R. tomentosa with those in other species. Fig S6. Distribution of synonymous substitution levels (Ks) of paralogous (A) and orthologous genes (B). Fig S7. Go analysis of positively selected genes in R. tomentosa. Fig S8. Chromosomal location of TPSs on chromosomes in R. tomentosa. [file 12864_2024_10509_MOESM1_ESM.pdf]
